# Intracerebral hemorrhage hospitalizations and outcomes: comparisons between institutional and national data

**DOI:** 10.1007/s11739-025-03977-5

**Published:** 2025-05-28

**Authors:** Chaitali Dagli, Zhuobin Huang, Chen Lin

**Affiliations:** 1 Department of Epidemiology, School of Public Health, University of Alabama at Birmingham, Birmingham, AL, USA; 2 Department of Neurology, University of Alabama at Birmingham, 1813 6th Avenue South, Birmingham, AL 35294, USA; 3 Birmingham VA Medical Center, Birmingham, AL, USA

**Keywords:** Intracerebral hemorrhage, In-hospital mortality, Length of stay, Regional disparities

## Abstract

Intracerebral hemorrhage (ICH) accounts for 10–15% of all strokes in the United States (US), with in-hospital mortality rates reaching 40–50%. This study addresses a critical gap in understanding ICH outcomes in Alabama, where data has historically been absent from the National Inpatient Sample (NIS), despite the state’s high burden of stroke incidence and mortality. To compare in-hospital mortality rates and length of stay (LOS) among patients with ICH treated at a comprehensive stroke center in Alabama versus large urban hospitals across the US, we conducted a retrospective analysis of data from 425 ICH patients treated at the academic comprehensive stroke center in Alabama (2016–2019) and 68,525 patients from the NIS (2016–2018). Patients were aged 18 and older with a diagnosis of ICH. We utilized multivariable logistic regression to assess mortality and linear regression for LOS, adjusting for demographics and procedures. The patients at the Alabama center were generally younger, white, and more likely to be females. The comprehensive stroke center reported an in-hospital mortality rate of 27.76 per 100 patients (95% CI = 22.76, 32.77), which was higher than the NIS average of 22.02 per 100 patients (95% CI = 21.69, 22.37). Additionally, patients at the Alabama center had 33.5% higher odds of in-hospital mortality compared to South Atlantic hospitals (OR = 1.34, 95% CI = 1.07−1.67, *p* = 0.0113). Lastly, the comprehensive stroke center demonstrated a significantly shorter LOS of 5.8 days (*β* = − 1.25; 95% CI = − 1.89, − 0.61; *p* = 0.0001) compared to the South Atlantic hospitals.

## Introduction

Intracerebral hemorrhage (ICH) is associated with high mortality and significant morbidity worldwide [1]. In the United States (US), approximately 10–15% of all strokes are attributed to ICH [2]. Furthermore, the in-hospital mortality rate for ICH remains alarmingly high, ranging from 40 to 50% [3]. Despite advancements in stroke care, outcomes such as in-hospital mortality and recovery metrics, including the length of stay (LOS) and the types of procedures performed, continue to vary widely across different regions and hospital types. A striking pattern of regional variation in stroke incidence and outcomes exists across the country. The southeastern US, often referred to as the“Stroke Belt,”having the highest rates of stroke incidence and mortality [4, 5]. Within this region, Alabama, Mississippi, and Tennessee are part of the“Stroke Buckle,”where the burden of stroke is especially pronounced [4]. Due to a combination of cardiovascular risk factors, sociodemographic disparities, and healthcare access issues, these states experience a disproportionate number of ICH cases [6, 7]. Alabama, in particular, faces a complex set of challenges in delivering equitable stroke care, given its higher prevalence of risk factors such as hypertension and diabetes.

A growing body of literature has highlighted the importance of hospital characteristics in shaping patient outcomes for ICH. Factors such as hospital size, geographical location, procedural capabilities, and patient population characteristics all play a role in determining mortality rates and LOS [8–12]. However, there is limited research that examines how institutions in the US South, particularly those in large urban areas, perform in comparison to hospitals in other regions. The treatment of ICH in large, specialized hospitals within major cities may offer insights into improving care at smaller or less resourced institutions, but it is unclear whether these outcomes can be replicated in settings that serve more diverse and vulnerable populations, such as those in Alabama.

A study by Khan et al. using the National Inpatient Sample (NIS) have provided valuable insights into national trends in in-hospital mortality, procedure types, and LOS for patients treated for ICH [13]. These data have been instrumental in identifying outcome disparities across various hospitals and urban centers. However, a notable gap exists due to the absence of data from Alabama—a state with high ICH incidence and unique demographic and healthcare characteristics. This study seeks to address this knowledge gap by comparing in-hospital mortality rates and LOS between a single major institution in Alabama and hospitals in large cities and urban centers across US. By focusing on the outcomes of patients treated for ICH, we aim to provide a clearer understanding of whether disparities exist between a large, specialized stroke center in Alabama and other regions. Our findings could offer key insights into optimizing treatment protocols and improving patient care for hemorrhagic stroke in Alabama, ultimately guiding regional strategies to reduce ICH mortality.

## Methods

### Data source

#### Academic comprehensive stroke center

Manual chart review on the ICH patients in the department of Neurology at the only academic comprehensive stroke center (Alabama center or academic center) located within Alabama was performed. The comprehensive chart review combined the medical record number with hospital patient record to extract additional variables such as demographic and clinical characteristics. Four hundred twenty-five patients were used from the registry from 2016 to 2019. Patients aged 18 or above who were diagnosed with ICH or transferred to hospital because of ICH were included in the database. The Institutional Review Board (IRB) of the academic center approved the data collection for and analysis of data using our institutional stroke registry [5, 14].

#### National Inpatient Sample (NIS)

The NIS dataset is the largest publicly available all-payer inpatients dataset in the US., sampling 20% of inpatient discharges from all US community hospitals. It currently contains data from more than seven million hospital stays each year in 48 states and the District of Columbia [15]. We used the NIS data between 2016 and 2018 and included 68,525 patients with ICH. The NIS dataset includes comprehensive information such as demographic characteristics, hospital characteristics, and outcome. All baseline characteristics of patients, hospital characteristics, diagnosis codes, and procedure codes were recorded in the dataset. The analysis of NIS data was considered exempt from review by the IRB.

The timeframe difference between the datasets (2016–2019 for the Alabama comprehensive center dataset vs. 2016–2018 for the NIS dataset) is primarily due to the data availability for each cohort. The NIS dataset includes 68,525 cases, while the Alabama dataset includes 425 cases. While the datasets cover slightly different timeframes, we believe this discrepancy does not significantly impact the comparison, as the overall trends in stroke care and outcomes are likely consistent across this short period.

### Inclusion and exclusion criteria

Patients aged 18 years or older, diagnosed with ICH, who were hospitalized between 2016 and 2019 in the academic stroke center data and between 2016 and 2018 in the NIS data, were included in the study. International Classification of Diseases, Tenth Revision, Clinical Modification (ICD-10-CM) code I61.0-I61.9 were used to select ICH patients from NIS data. Patients whose discharge disposition was transferred out in the NIS dataset were excluded.

### Variables

#### Primary independent variable

Our primary independent variable was the hospital area subgroup variable in the combined NIS and academic comprehensive stroke center datasets. The subgroups in the NIS dataset were stratified by variables originally collected in the NIS dataset: hospital bed size (small/medium/large), location/teaching status of hospital (rural/urban non-teaching/urban teaching), region of hospital (Northeast/Midwest/South/West), and census division of hospital (New England/Middle Atlantic/East North Central/West North Central/South Atlantic/East South Central/West South Central/Mountain/Pacific). We restricted the NIS data to ICH patients in large, urban teaching hospitals in the South, and then stratified by census divisions: South Atlantic, East South Central, and West South Central. After stratification, we created a variable with four levels of census divisions (South Atlantic Large Urban Teaching/East South Central Large Urban Teaching/West South Central Large Urban Teaching/academic comprehensive stroke center). A second variable was created by combining the academic comprehensive stroke center dataset and East South Central Large Urban Teaching Hospital into a single category, resulting in a 3-level census division variable with South Atlantic Large Urban Teaching Hospitals and West South Central Large Urban Teaching Hospitals as the other two levels.

### Primary outcomes

Our primary outcomes were in-hospital mortality and length of stay (LOS).

### Covariates

Sociodemographic variables such as Age (18–44/45–64/65–84/85 +), gender (male/female), race and ethnicity (White/Black or African American/Asian/Other) were included in the study. We further included insurance and procedures (clot evacuation/decompression included craniotomy, craniectomy, cranioplasty, burr hole, clot evacuation, clot aspiration, Hydrocephalus-related included extra ventricular device (EVD) and ventriculoperitoneal shunting (VPS) or repair of vascular malformations included clipping, coiling, venous thrombectomy, arteriovenous malformation (AVM) embolization and AVM resection). Procedures in the NIS dataset were selected and coded using the International Classification of Diseases, Tenth Revision, Procedure Coding System (ICD-10-PCS). Lastly, death was included as a covariate in the analysis of the association between census divisions and LOS in the adjusted linear regression models.

### Statistical analysis

Descriptive statistics were generated for baseline demographic and clinical characteristics across the four groups: academic comprehensive stroke center, South Atlantic large urban teaching hospitals, East South Central large urban teaching hospitals, and West South Central large urban teaching hospitals. Continuous variables were analyzed using one-way analysis of variance (ANOVA), while categorical variables were analyzed using Pearson’s chi-square test or Fisher’s exact test, where appropriate.

To assess the association between hospital area and in-hospital mortality, we performed multivariable logistic regression, adjusting for age, gender, race, insurance, and procedures. Separate models were created for the academic comprehensive stroke center data and each of the NIS subgroups (South Atlantic, East South Central, and West South Central large urban teaching hospitals). A final combined model included data from academic comprehensive stroke center and the NIS South Large Urban Teaching Hospitals, using the four-level hospital area variable (South Atlantic large urban teaching, East South Central large urban teaching, West South Central large urban teaching, and academic comprehensive stroke center). The odds ratios (ORs) and corresponding 95% confidence intervals (CIs) were calculated for the association between each hospital area and in-hospital mortality, using South Atlantic large urban teaching hospitals as the reference group.

To examine the association between hospital area and LOS, we used multivariable linear regression, adjusting for age, gender, race, insurance, procedures, and in-hospital death. Linear regression models were built separately for each hospital area and a combined model was created using the four-level hospital area variable. Patients with a hospital stay longer than 30 days were excluded from the analysis to reduce skewness in LOS distribution. Beta coefficients (*β*) and corresponding 95% CIs were calculated for each hospital area, with South Atlantic large urban teaching hospitals as the reference group. The linear regression analysis assessed whether the LOS differed significantly between academic comprehensive stroke center, East South Central, and West South Central large urban teaching hospitals.

All statistical analyses were performed using SAS version 9.4. A two-sided *p*-value of < 0.05 was considered statistically significant.

## Results

Table 1 presents the demographic and procedures of patients from academic comprehensive stroke center and three large urban teaching hospital subgroups in the South (South Atlantic, East South Central, and West South Central). Across the groups, significant differences were observed in age distribution (*p* = 0.0004), with the majority of patients aged 45–84. Racial composition varied significantly (*p* < 0.0001), with academic center having a higher percentage of Black patients (42.82%) compared to other regions. Insurance coverage also differed significantly (*p* < 0.0001), with Medicare being the most common insurance type. Furthermore, the academic center had higher rates of clot evacuation/decompression (7.29%) and hydrocephalus-related procedures (12.00%) compared to other hospitals. Significant differences were also observed for vascular malformation repair (*p* = 0.0005) and LOS (*p* = < 0.001).

**Fig. 1:**
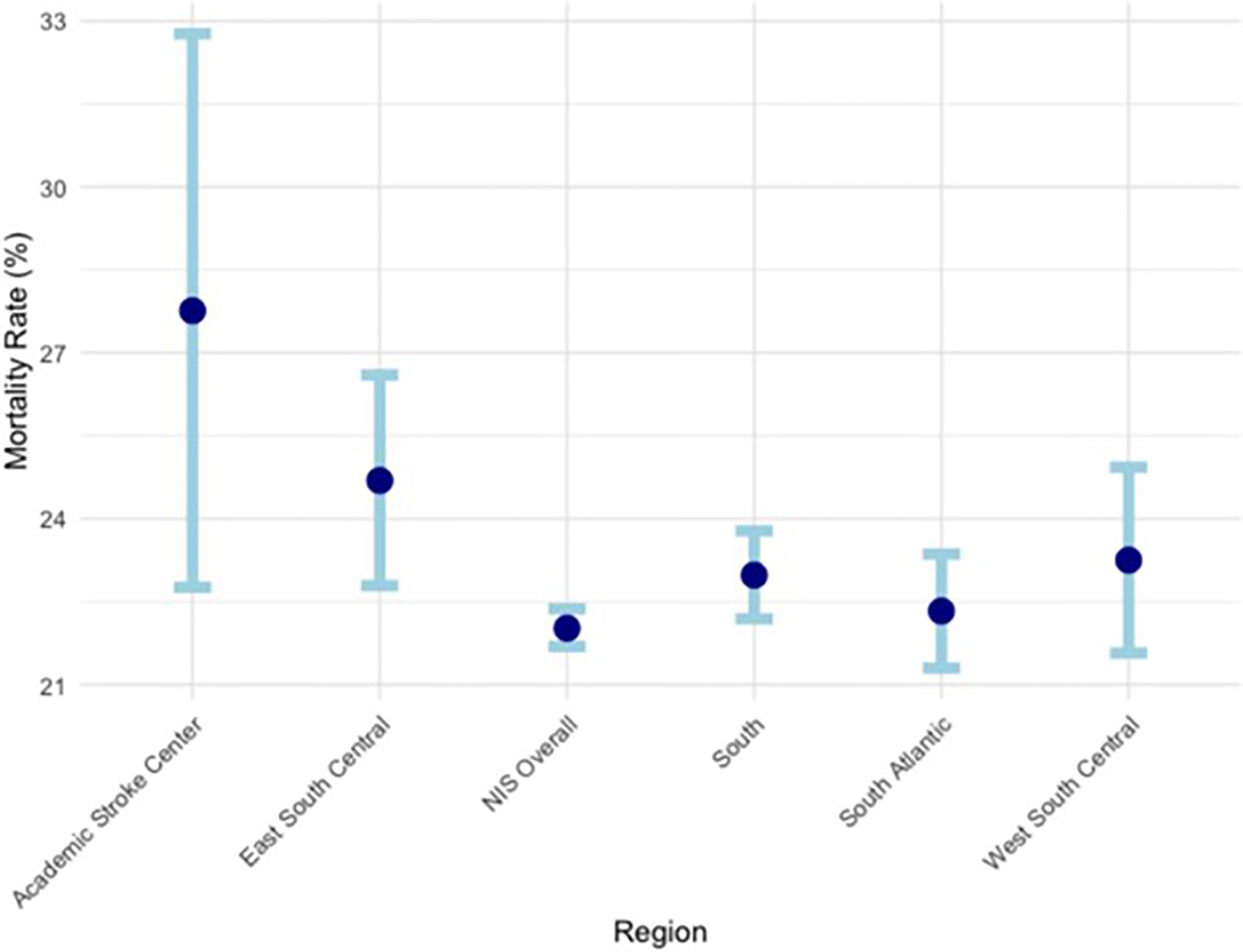
In-hospital mortality rates

Table 2 compares the in-hospital mortality rates (per 100 patients) between academic comprehensive stroke center and different areas of the South based on NIS data. The academic comprehensive stroke center had a higher in-hospital mortality rate of 27.76 per 100 patients (95% CI = 22.76, 32.77) compared to the overall NIS mortality rate of 22.02 per 100 patients (95% CI = 21.69, 22.37) (Fig. 1). Within the NIS South region, the mortality rate was slightly higher at 22.31 per 100 patients (95% CI = 21.75, 22.87). When further stratified into large urban teaching hospitals, the academic center’s mortality rate remained higher than the South overall (22.98 per 100 patients, 95% CI = 22.19, 23.78), East South Central (24.69 per 100 patients, 95% CI = 22.79, 26.60), and West South Central (23.25 per 100 patients, 95% CI = 21.57, 24.93). The East South Central region, which includes academic center’s location, had the highest mortality rate among the large urban teaching hospital subgroups.

Table 3 presents the results of a logistic regression analysis assessing the association between hospital area and in-hospital mortality, adjusted for age, gender, race, insurance, and procedures. Patients in academic comprehensive stroke center (OR = 1.34, 95% CI = 1.07, 1.67, *p* = 0.0113) and East South Central large urban teaching hospitals (OR = 1.14, 95% CI = 1.03, 1.27, *p* = 0.0136) had a significantly higher odds of in-hospital mortality compared to South Atlantic large urban teaching hospitals.

Table 4 shows the results of a linear regression assessing the association between hospital area and LOS in academic comprehensive stroke center and other large urban teaching hospitals in different regions of the South, adjusted for covariates. For the academic comprehensive stroke center, after excluding patients with a hospital stay longer than 30 days to reduce skewness in the LOS distribution, 392 patients remained for final analysis. The academic comprehensive stroke center had a significantly shorter LOS (*β* = − 1.25; 95% CI = − 1.89, − 0.61; *p* = 0.0001) compared to South Atlantic large urban teaching hospitals. Similarly, East South Central large urban teaching hospitals also had a shorter LOS (*β* = − 0.32, 95% CI = − 0.61, − 0.03; *p* = 0.0281). However, the difference in LOS for West South Central large urban teaching hospitals was not statistically significant (*β* = − 0.11, 95% CI = − 0.38, 0.17; *p* = 0.4438).

## Discussion

This study examined in-hospital mortality rates and LOS among patients with ICH between other similar hospitals and regions using the NIS data compared to the largest and only comprehensive stroke center in Alabama. The NIS includes data from 48 states, with Alabama, Nevada, and Idaho not participating due to state-specific policy and funding decisions [16]. The exclusion of Alabama data from the NIS dataset is a significant limitation, as this state experiences some of the highest rates of stroke incidence and mortality in the nation. This gap in data restricts the ability to understand the true impact of regional healthcare disparities on stroke outcomes. By incorporating data from Alabama’s only comprehensive stroke center, our study fills an important gap, allowing for a more accurate comparison of ICH outcomes. This addition not only highlights the specific challenges faced by patients in Alabama but also underscores the need for targeted strategies to improve stroke care in the Southeast, where the burden of disease is particularly heavy.

The results of our study highlight notable differences in ICH outcomes between patients treated at this academic comprehensive stroke center and those treated in other large urban hospitals in the US. We found that the Alabama center and hospitals in the East South Central region had higher in-hospital mortality compared to South Atlantic hospitals. While mortality rates at the academic comprehensive stroke center were slightly higher, this may reflect underlying regional health disparities, especially given the elevated prevalence of risk factors such as hypertension, diabetes, and unique racial demographics in Alabama and the broader Southeast region [17–19]. In addition, we observed a relatively small proportion of patients aged 85 years and older at the Alabama center (8.47%), which is lower than what is typically seen in studies of ICH. This may be due to lower referral rates for older patients, possibly reflecting concerns over the futility of treatment or fragility of this age group, which could lead to fewer transfers to the comprehensive stroke center. In contrast, both the Alabama center and East South Central hospitals had shorter LOS than their South Atlantic counterparts.

We found that the rate of surgical interventions and advanced procedures, such as craniotomy and EVD placement, was higher at the Alabama center compared to some national averages. This increased rate may reflect the center’s status as a comprehensive academic stroke center, equipped with 24-h access to dedicated vascular neurosurgeons and serving a large referral base across Alabama, including rural communities where access to specialized neurological care is limited [20]. The slightly higher in-hospital mortality rate for ICH patients at our center compared to national urban averages could similarly stem from the complex cases referred to our center from underserved areas. These findings are consistent with previous studies showing that stroke outcomes tend to be worse in the Stroke Belt compared to other parts of the US [4, 19, 21, 22]. For example, Howard et al. (2017), reported that mortality from both ischemic and hemorrhagic stroke is significantly higher in the southeastern U.S. [19]. Similarly, Thompson et al. (2022) demonstrated that despite improvements in stroke care nationwide, regions with poorer access to specialized neurological services still experience worse outcomes for acute stroke interventions, which may be partly responsible for the differences observed in our study [21].

Patients at the academic comprehensive stroke center had a longer average LOS than their counterparts treated in large hospitals nationally. This finding could be explained by several factors, including the higher severity of illness among patients admitted at the academic comprehensive stroke center and potential delays in post-acute care transfers, which have been well documented in the Southeast [23]. Previous studies have shown that hospitals in regions with lower access to rehabilitation and post-acute services often experience delays in discharging stroke patients, leading to prolonged hospital stays and higher costs of care [11, 12, 20]. A study by Ikeme et al. (2022) highlighted how socioeconomic and healthcare system disparities could contribute to delays in stroke recovery and post-acute care, potentially leading to extended LOS and suboptimal recovery outcomes [24]. The longer LOS observed at the academic comprehensive stroke center, therefore, may be due to systemic issues, such as difficulties in accessing rehabilitation services or transferring patients to long-term care facilities. Findings from a recent study using registry data on ICH patients in rehabilitation settings observed that longer LOS is more common at academic centers, particularly within the Stroke Belt [14]. This region which consistently exhibits higher rates of stroke incidence and mortality compared to national averages [4, 19], may face exacerbated disparities due to a high burden of cardiovascular risk factors alongside systemic challenges like limited access to specialized stroke care. These regional disparities likely contribute to the poorer outcomes observed in this area.

The higher in-hospital mortality rates in the Southeast emphasize the need for more comprehensive stroke care strategies in this region. With hypertension and diabetes rates being especially high, early identification and management of these conditions are vital for improving outcomes among patients at risk for ICH. Moreover, ICH disproportionately affects Black patients [25, 26], highlighting a significant health disparity linked to both a higher prevalence of risk factors and uneven access to advanced care. Expanding access to specialized stroke services and reducing delays in rehabilitation could help address these disparities to improve recovery rates and reduce mortality in underserved communities across the Southeast.

This study has several limitations. First, as a comprehensive stroke center, this hospital likely receives more severe cases requiring advanced interventions, which may contribute to higher mortality rates. While we compared outcomes with large urban teaching hospitals, not all of these are comprehensive stroke centers, which may affect the generalizability of our findings. Additionally, the NIS dataset does not identify specific hospitals, making it difficult to directly compare outcomes from other comprehensive stroke centers nationwide, and the exclusion of states like Alabama, Nevada, and Idaho from the NIS dataset further limits national data generalizability. Moreover, the NIS does not include data on clinical severity, so we were unable to control for in our analysis. Furthermore, for the NIS dataset, we no longer have access to the data, and as a result, we are unable to provide the number of patients excluded based on length of stay (LOS). Lastly, discrepancies in data availability between the two datasets, particularly regarding clinical severity may introduce information bias, but we do not believe it significantly influenced the interpretation of our results.

Despite these limitations, our study has several key strengths. By utilizing data from a major academic center in Alabama with a stroke registry, we conducted a detailed analysis of in-hospital mortality rates and LOS for ICH patients. Comparing outcomes at this specialized center with NIS data addresses a critical gap in the literature, particularly given Alabama’s high stroke incidence and unique demographic and healthcare challenges. The inclusion of various clinical factors also allows for a comprehensive assessment of influences on ICH outcomes, shedding light on patient care variables often overlooked. While our analysis relies on retrospective data, which could introduce variations in coding practices, focusing on an academic center in a large urban area provides valuable insights into outcomes for patients treated in high-volume facilities.

## Conclusion

In conclusion, our study highlights significant regional and institutional disparities in ICH outcomes between the academic stroke center and other large hospitals in the US. The findings point to the complex interplay of patient demographics, risk factors, and healthcare infrastructure in determining stroke outcomes. By addressing these disparities and enhancing stroke care delivery in underserved regions, there is potential to reduce the burden of ICH in Alabama and beyond.

## Figures and Tables

**Table 1:** Demographic characteristics*

Characteristics	Academic comprehensive stroke center *N* = 425	South Atlantic Large Urban teaching *N* = 8088	East South Central Large Urban teaching *N* = 2612	West South Central Large Urban teaching *N* = 3170	*p*-value
Demographic characteristics					
Age in years, *n* (%)					0.0004^‡^
18–44	62 (14.59)	750 (9.27)	246 (9.42)	338 (10.66)	
45–64	151 (35.53)	2882 (35.63)	916 (35.07)	1204 (37.98)	
65–84	176 (41.41)	3562 (44.04)	1167 (44.68)	1328 (41.89)	
85 +	36 (8.47)	894 (11.05)	283 (10.83)	300 (9.46)	
Gender, *n* (%)					0.9537
Male	205 (48.24)	4202 (51.95)	1343 (51.42)	1653 (52.15)	
Female	220 (51.76)	3886 (48.05)	1269 (48.58)	1517 (47.85)	
Race, *n* (%)					<.0001^‡^
White	221 (52.00)	4843 (59.88)	1994 (76.34)	1574 (49.65)	
Black	182 (42.82)	2443 (30.21)	555 (21.25)	587 (18.52)	
Asian	12 (2.82)	152 (1.88)	11 (0.42)	80 (2.52)	
Other	10 (2.35)	650 (8.04)	52 (1.99)	929 (29.31)	
Insurance, *n* (%)					<.0001^‡^
Medicare	218 (51.29)	4389 (54.27)	1590 (60.87)	1663 (52.46)	
Medicaid	54 (12.71)	846 (10.46)	262 (10.03)	248 (7.82)	
Private	110 (25.88)	1999 (24.72)	505 (19.33)	760 (23.97)	
Other	42 (10.12)	854 (10.56)	255 (9.76)	499 (15.74)	
LOS, mean (SD)	7.35 (5.85)	8.18 (6.90)	7.53 (6.46)	8.08 (6.83)	<.0001^‡^
Procedures					
^†^Clot evacuation/decompression	31 (7.29)	42 (0.52)	5 (0.19)	23 (0.73)	<.0001^‡^
^†^Hydrocephalus-related	51 (12.00)	786 (9.72)	188 (7.20)	306 (9.65)	0.0002^‡^
^†^Repair of vascular malformations	5 (1.18)	399 (4.93)	148 (5.67)	140 (4.42)	0.0005^‡^

* One-way analysis of variance (ANOVA) was used for continuous variables, and Pearson’s chi-square test or Fisher’s exact test was used for categorical variables

† Clot evacuation/decompression include craniotomy, craniectomy, cranioplasty, burr hole, clot evacuation, clot aspiration); hydrocephalus-related include EVD, VPS; repair of vascular malformations include clipping, coiling, venous thrombectomy, AVM embolization, and AVM resection

‡ Significant *p*-values

**Table 2:** In-hospital mortality rate of academic comprehensive stroke center and different areas of south

Variable	Overall	South	South Atlantic	East South Central	West South Central
Comprehensive stroke center	27.76 (22.76, 32.77)				
NIS	22.02 (21.69, 22.37)	22.31 (21.75, 22.87)			
Large urban teaching	21.69 (21.22, 22.16)	22.98 (22.19, 23.78)	22.33 (21.30, 23.36)	24.69 (22.79, 26.60)	23.25 (21.57, 24.93)

* Adjusted for age, gender, race, insurance, and procedures

**Table 3:** Multivariable logistic regression to assess the association between hospital area and in-hospital mortality

Variable	Mortality		
	OR	95% CI	*p*-value
South Atlantic large urban teaching	Ref		
East South Central large urban teaching	1.14	1.03, 1.27	0.0136^†^
West South Central large urban teaching	1.05	0.95, 1.16	0.3559
Academic comprehensive stroke center	1.34	1.07, 1.67	0.0113^†^

* Adjusted for age, gender, race, insurance, and procedures

† Significant *p*-values

**Table 4:** Linear regression to assess the association between hospital area and LOS

Variable	*β*	95% CI	*p*-value
South Atlantic large urban teaching	Ref		
East South Central large urban teaching	− 0.32	− 0.61, − 0.03	0.0281^†^
West South Central large urban teaching	− 0.11	− 0.38, 0.17	0.4438
Academic comprehensive stroke center	− 1.25	− 1.89, − 0.61	0.0001^†^

* Adjusted for age, gender, race, insurance, death, and procedures

† Significant *p*-values

## Data Availability

The data from the Alabama comprehensive stroke center registry are not publicly available due to institutional restrictions but may be made available from the corresponding author on reasonable request and with appropriate institutional approvals. The NIS database is publicly available and can be accessed through the Healthcare Cost and Utilization Project (HCUP) at https://www.hcup-us.ahrq.gov/nisoverview.jsp.
